# Germinated Spores of the Probiotic Bacterium *Bacillus coagulans* JBI-YZ6.3 Support Dynamic Changes in Intestinal Epithelial Communication and Resilience to Mechanical Wounding

**DOI:** 10.3390/microorganisms13071466

**Published:** 2025-06-24

**Authors:** Sage V. McGarry, Earvin A. F. Grinage, Krista Sanchez, Dina Cruickshank, Liang Anderson, Gitte S. Jensen

**Affiliations:** 1 NIS Labs, 807 St. George St., Port Dover, ON N0A 1N0, Canada; sage@nislabs.com (S.V.M.); dina@nislabs.com (D.C.); 2 NIS Labs, 1437 Esplanade, Klamath Falls, OR 97601, USA; earvin@nislabs.com (E.A.F.G.); krista@nislabs.com (K.S.); liang@nislabs.com (L.A.)

**Keywords:** anti-inflammatory, chemotactic recruitment, IL-6, IL-8, IP-10, MIP-1α, MIP-1β, wound healing, zonulin

## Abstract

The spore-forming probiotic *Bacillus coagulans* JBI-YZ6.3 interacts with the gut epithelium via its secreted metabolites as well as its cell walls, engaging pattern-recognition receptors on the epithelium. We evaluated its effects on human T84 gut epithelial cells using in vitro co-cultures, comparing metabolically active germinated spores to the isolated metabolite fraction and cell wall fraction under unstressed versus inflamed conditions. Germinated spores affected epithelial communication via chemokines interleukin-8, interferon gamma-induced protein-10, and macrophage inflammatory protein-1 alpha and beta after 2 and 24 h of co-culture. Non-linear dose responses confirmed that bacterial density affected the epigenetic state of the epithelial cells. In contrast, the cell wall fraction increased cytokine and chemokine levels under both normal and inflamed conditions, demonstrating that the intact bacterium had anti-inflammatory properties, regulating pro-inflammatory signals from its cell walls. During recovery from mechanical wounding, germinated spores accelerated healing, both in the absence and presence of LPS-induced inflammation; both the metabolite and cell wall fractions contributed to this effect. The release of zonulin, a regulator of tight junction integrity, was reduced by germinated spores after 2 h. These findings suggest that *B. coagulans* JBI-YZ6.3 modulates epithelial chemokine signaling, supports barrier integrity, and enhances epithelial resilience, highlighting its potential as an efficacious multi-faceted probiotic for gut health.

## 1. Introduction

The microbiome of the gastrointestinal tract directly interacts with the human host and affects host health in a profound manner [1], including the digestion of nutrients [2,3,4], modulation of the immune system [5], and interfacing with the nervous system via the gut–brain axis [6]. Dysbiosis involves disruption of the microbial ecosystem [7,8], with changes to the functional composition and metabolic activities leading to a loss of biodiversity and functional capacity.

**Figure 1:**
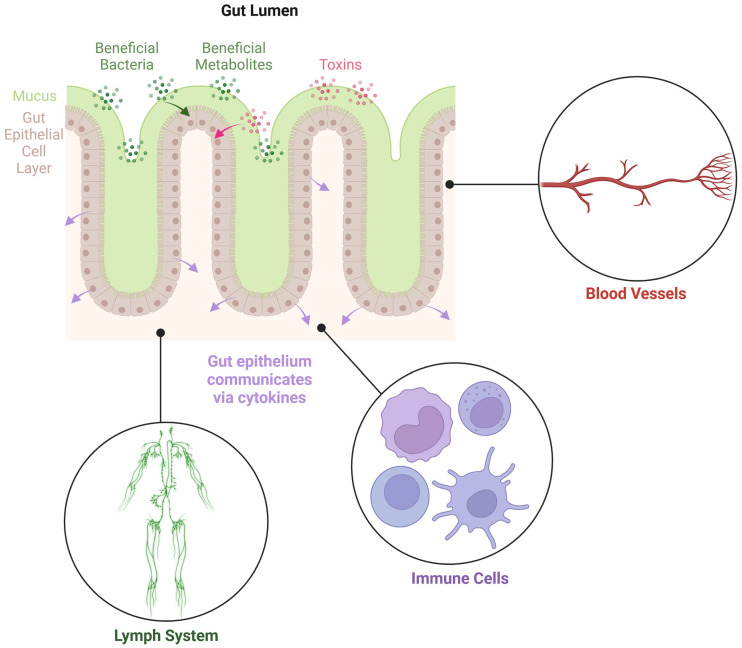
Diagram illustrating the gut epithelial cell layer, separating the gut lumen from the underlying tissues. The epithelial cells play pivotal roles in communication between the gut microbiome and the host.

Emerging evidence suggests that the microbiome’s metabolomic output is more impactful than its specific taxonomic composition [9,10,11,12,13]. Certain bacteria are associated with the regulation of gut inflammation [14] and have the capacity to produce beneficial metabolites such as butyrate [15], while other harmful bacteria produce metabolites that are stressful to the host [16]. Microbial metabolites from both beneficial and pathogenic microbes are absorbed into underlying immunologically active tissue, disseminated via the lymphatic and blood circulation, and can be detected in the blood circulation of the host (Figure 1) [5].

**Figure 2:**
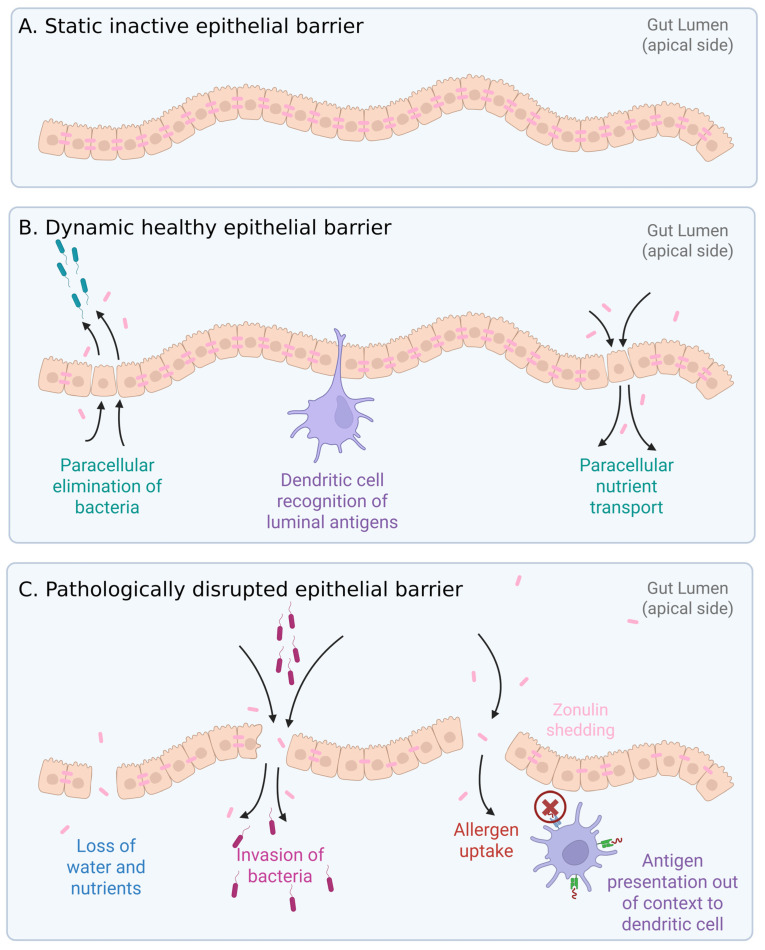
Gut epithelial structure and function. Zonulin plays an active regulating role in tight junctions. (**A**) Static epithelial barrier. (**B**) Healthy epithelial barrier, where dynamic changes to tight junctions allow paracellular elimination of invading bacteria, immune cell scavenging of luminal antigens, and paracellular transport of nutrients. (**C**) Pathologically disrupted barrier where nutrients are leaking, and opportunistic bacteria penetrate across the barrier. Allergens (environmental and food-related) can cross the barrier. Both bacterial and allergen antigens can elicit inappropriate immune responses by presentation to dendritic cells out of context.

Microbial and human cells interact extensively along the intestinal mucosal barrier, wherein a monolayer of epithelial cells connected by tight junctions forms a physical barrier protecting the host from the external environment while facilitating nutrient absorption [17]. Tight junctions provide a paracellular pathway for selective transportation of small-molecule nutrients, the flux of bacterial compounds, and controlled mucosal antigen trafficking, leading to mucosal tolerance [18]. Zonulin, a physiological regulator of tight junctions, can reversibly disassemble tight junctions and increase the permeability of paracellular space, playing a key role in nutrient absorption while maintaining the integrity of tight junctions and mucosal homeostasis [19,20]. Some pathological conditions, such as Celiac disease and infections, can trigger an increase in zonulin expression and release from the epithelium, thereby causing an uncontrolled increase in intestinal permeability and mucosal barrier dysfunction [21]. Further, pathological levels of zonulin shedding can lead to increased trafficking of antigens and pathogens into mucosal tissue and initiate abnormal local or systemic immune responses [22] (Figure 2).

Probiotic bacteria are live microorganisms that confer health benefits to the host [23]. There are many different genera of probiotic bacteria, including *Lactobacillus*, *Akkermansia*, *Bifidobacterium*, and *Bacillus* [24]. Probiotic bacteria are effective in preventing and treating gastrointestinal disorders, including inflammatory bowel disease and antibiotic-associated diarrhea [25,26,27,28], while also influencing metabolic health and cognitive function [29]. Probiotic bacteria face significant challenges in surviving extreme conditions during manufacturing and in the harsh environment of the gut, and therefore certain species may not maintain viability to confer optimal health benefits to the host [30,31].

Spore-forming *Bacillus* species, including *Bacillus coagulans*, are ideal candidates for functional foods [32,33,34,35,36] that repair intestinal permeability by supporting healthy tight junction protein production [37]. *B. coagulans* positively influences the diversity and metabolic function of intestinal and vaginal microbiota [38], offering therapeutic benefits by modifying the host’s microbiota and thereby the metabolomic capacity of the microbiome [39]. *B. coagulans* secretes metabolites with immune-activating and modulating properties [40], including anti-inflammatory effects [41], maturation of antigen-presenting cells [42], and cytotoxic elimination of transformed target cells [41]. Furthermore, the secreted metabolites from *B. coagulans* JBI-YZ6.3 trigger increased production of anti-inflammatory markers in cultures of peripheral blood mononuclear cells [40].

This immunomodulating effect by *B. coagulans*-secreted metabolites warranted further work, specifically to document effects on gut epithelial cells. In the present study, we used the T84 human epithelial colorectal adenocarcinoma cell line, widely used as an in vitro model of gut epithelial function, viability, integrity, wound healing, and cytokine secretion [43]. T84 cells are highly dynamic and have the ability to exhibit immunostimulatory characteristics, both by communication via cytokines and growth factors, and by formation of exosome vesicles that perform antigen-presenting functions [44]. We cultured T84 cells in the presence of *B. coagulans* JBI-YZ6.3 germinated spores, metabolite fraction, and cell wall fraction, in the absence versus presence of inflammation, and we evaluated the effects of the probiotic on wound recovery and zonulin release. The ability of this probiotic bacterium to regulate epithelial cell production of selective chemokines under unstressed versus inflamed conditions is significant in the advancement of probiotic potential for rapid and sustained support of gut health and communication to the immune system.

## 2. Materials and Methods

### 2.1. Reagents

Dulbecco’s modified Eagle’s medium with nutrient mixture F-12 (DMEM F-12) with phenol red (catalog # 11320-033) and without phenol red (catalog # 11039-021), penicillin–streptomycin 100× (Gibco catalog # 15140-122), Roswell Park Memorial Institute 1640 medium (Gibco catalog # 11835-030), fetal bovine serum (Gibco catalog # A38401-01), Dulbecco’s phosphate-buffered saline (Gibco catalog # 141190-136), lipopolysaccharide (LPS) (Invitrogen catalog # 00-4976-93), and TrypLE Express Enzyme (catalog # 12604013) were purchased from Thermo Fisher Scientific Inc. (Waltham, MA, USA). Bio-Plex Pro™ human cytokine arrays were purchased from Bio-Rad Laboratories, Inc. (Hercules, CA, USA). Zonulin ELISA kits (catalog # ABIN6962693) were purchased from antibodies-online Inc. (Limerick, PA, USA).

### 2.2. B. coagulans Germinated Spores

*B. coagulans* JBI-YZ6.3 spores were provided by the study sponsor, Jeneil Biotech, Inc. (Saukville, WI, USA). The strain is deposited as ATCC PTA-127366 at the American Type Culture Collection. The genome sequence of the bacterium [45] is available from GenBank (CP104390), and no potential antibiotic resistance genes or toxin genes have been detected in this strain [46].

Spores were prepared via aseptic fermentation as previously described [40]. Briefly, proprietary media and fermentation parameters resulted in a cell population comprised primarily of endospores. The endospores were harvested by centrifugation and lyophilized in the absence of cryoprotectant. Dried biomass was standardized to a concentration of 1.5 × 10^10^ colony-forming units/gram by blending with identity-preserved maltodextrin.

*B. coagulans* JBI-YZ6.3 germinated spores were freshly prepared before each cell culture, as previously described [40]. In brief, a sample of dry spores was added to sterile PBS (40 mg/mL) and mixed until a uniform suspension was generated. The spore suspension was incubated for 30 min at room temperature to allow the spores to hydrate, after which time spore solutions were sonicated for 10 min to reduce the number of aggregated spores. The suspension was transferred to a preheated water bath at 80 °C and incubated for 20 min. After incubation, the spore suspension was cooled immediately to 45 °C with intermittent vigorous agitation. This suspension was used to prepare serial dilutions for addition to cell cultures.

### 2.3. B. Coagulans Metabolites and Cell Wall Fractions

A sample of germinated spores was cultured in Roswell Park Memorial Institute 1640 (RPMI-1640) culture medium under aerobic culture conditions at 37 °C as described previously [40], where the resulting culture was used to prepare both the metabolite fraction and the cell wall fraction for testing.

Metabolite fraction: The supernatant containing the fermentation metabolites was harvested by centrifugation and sterile-filtered using a 0.22 μm cellulose acetate filter, resulting in no detectable colony-forming units in this fraction. Multiple aliquots were frozen at −30 °C.

Cell wall fraction: The bacteria were pelleted by centrifugation, and washed three times in PBS, followed by three freeze–thaw cycles. After the freeze–thaw cycles, the bacterial cell walls were broken via bead-milling using low-protein binding 100-micron zirconium beads and ten cycles of sixty 1 s pulses by vigorous vortexing, with each cycle followed by 30 s immersion into an ice bath. After the beads had settled, the supernatant containing the cell wall material was transferred to a clean microcentrifuge tube. Cell wall fractions were pelleted by centrifugation and the supernatant was discarded. Final purified cell wall fractions were resuspended in sterile PBS. Multiple aliquots were frozen at −30 °C.

### 2.4. Gut Epithelial Cell Cultures

T84 cell cultures were established in Dulbecco’s modified Eagle’s medium with nutrient mixture F-12 (DMEM F-12), allowing the cells to differentiate into mature cultures where the cells formed confluent layers connected by tight junctions. Fully differentiated T84 cells were harvested and transferred into 24-well plates using the following procedure: cells were washed twice in PBS, detached at 37 °C for 10 min using TrypLE (Gibco, Waltham, MA USA), and counted using a hemocytometer and Trypan Blue dye exclusion to ensure live cells were counted. The cell density was adjusted to 60,000 cells per mL, with 1 mL per well. The cells were cultured in DMEM-F-12 with phenol red, 10% fetal calf serum, penicillin (100 units/mL, where one unit is 0.6 μg), and streptomycin (100 μg/mL), except in zonulin supernatant experiments, where DMEM F-12 without phenol red or fetal calf serum was used.

### 2.5. Metabolic Activity of Germinated Spores

**Figure 3:**
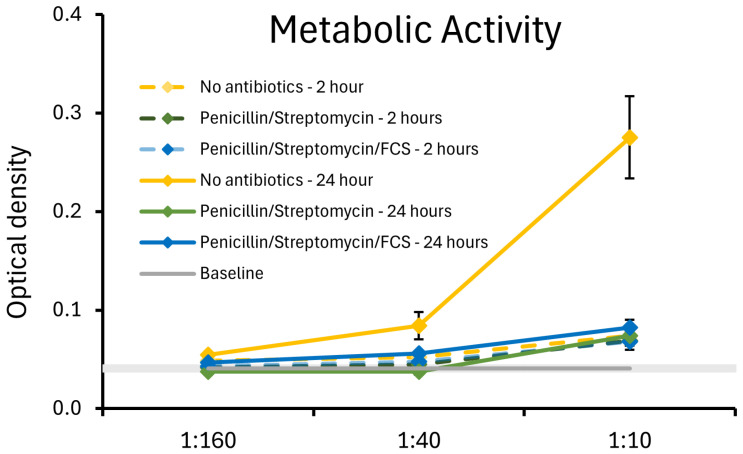
Relative metabolic activity of the germinated spores of *B. coagulans* JBI-YZ6.3 in the absence versus presence of penicillin, streptomycin, and fetal calf serum (FCS), mimicking the cell culture conditions used for the experimental testing in co-cultures of *B. coagulans* with T84 human gut epithelial cells. At 24 h, the metabolic activity of *B. coagulans* treated with antibiotics was statistically significantly lower than the cultures without antibiotics (*p* < 0.05). For the 1:40 and 1:160 dilutions, the metabolic activity of cultures in the presence of fetal calf serum was significantly higher than that of cultures treated with penicillin and streptomycin in the absence of fetal calf serum (*p* < 0.01).

The strain JBI-YZ6.3 was previously shown to be susceptible to multiple antibiotics, whereas under the culture conditions described here, we observed metabolic activity but only minimal cell division. The downstream co-cultures of *B. coagulans* JBI-YZ6.3 and T84 epithelial cells were performed in the presence of antibiotics to prevent bacterial overgrowth in the co-cultures. Therefore, it was an important validation of our methodology to document the metabolic activity of *B. coagulans* in the absence versus the presence of antibiotics. The metabolic activity of the germinated spores was tested under the same culture conditions as in the T84 co-cultures using the MTT assay, a colorimetric method that utilizes the tetrazolium dye 3-(4,5-di-Methyl-Thiazol-2-yl)-2,5-diphenyl-Tetrazolium bromide (MTT), which is converted into an insoluble purple compound, formazan. Each dose was tested in triplicate. After 2 and 24 h, the MTT dye was added, and incubated for 1.5 h to allow the formation of formazan crystals. Subsequently, sodium dodecyl sulfate was added to dissolve the formazan crystals, and the color intensity was measured at 570 nanometers in a spectrophotometer plate reader (PowerWave X340, BioTek Instruments, Winooski, VM, USA). Using DMEM F-12 culture medium, three culture conditions were compared: (1) no antibiotics; (2) penicillin and streptomycin; and (3) penicillin, streptomycin, and 10% fetal calf serum. The metabolic activity at 2 and 24 h was documented (Figure 3). In the absence of antibiotics, bacterial cell division was obvious after 24 h. In the presence of antibiotics, there was metabolic activity at both 2 and 24 h, but only a marginal increase from 2 to 24 h, suggesting a very low level of cell division compared to the bacterial cultures without antibiotics. The presence of fetal calf serum had a mild protective effect on the bacterial metabolic activity under antibiotic treatment, and the metabolic activity at 24 h for germinated spores treated with penicillin, streptomycin, and fetal calf serum was statistically significant compared to baseline for all doses of the germinated spores.

### 2.6. Treatment of T84 Cells with Germinated Spores, Metabolite Fraction, and Cell Wall Fraction

Treatment with germinated spores: Serial dilutions of germinated spores (1:10, 1:40, and 1:160, based on previous work [40]) were added to the T84 cultures, where each dose of germinated spores was tested in triplicate for each culture condition. Germinated spores were diluted, so that the doses delivered to T84 cultures were equivalent to 0.025, 0.100, and 0.400 mg/mL of the initial dried biomass, equating to 0.375, 1.5, and 6.0 million germinated spores per mL cell culture, respectively. Untreated epithelial cells were used as a negative control.

Treatment with metabolite fraction and cell wall fraction: One aliquot of each metabolite fraction and cell wall fraction was thawed on each lab testing day, vortexed, and used for testing in T84 cell cultures. The final doses in the T84 cultures were 1:10-, 1:40-, and 1:160-fold dilutions of the initial frozen stock solutions.

### 2.7. Mechanical Wounding and Wound Recovery

Mechanical wounding of T84 cells was achieved by performing a scrape across the tight monolayer of T84 cells with a sterile pipette tip and allowing the cells to rest for 2 h. After 2 h, the wells were washed once in sterile PBS to remove any cells and debris created during the mechanical wounding, after which fresh DMEM F-12 medium and test products were added. Subsequently, 10 min after adding fresh medium and test products, LPS (5 μg/mL) was added to half the wells to induce inflammation. Mechanically wounded cultures in the absence of test products and LPS served as negative controls, and LPS-treated cultures served as positive controls. Mechanically wounded T84 cultures were examined visually for 5 days to monitor recovery, once per day. Repair was compared for product-treated cells versus untreated cells under normal and LPS-inflamed culture conditions. After 5 days, the recovery of each condition in triplicate was scored on a scale from 0 to 10, where “0” indicated no recovery, and “10” indicated complete recovery of the confluent cell layer.

### 2.8. Zonulin Release

Zonulin shedding was evaluated using a commercial enzyme-linked immunosorbent assay (ELISA) kit (antibodies-online Inc. catalog # ABIN6962693, Limerick, PA, USA), following the protocol provided by the manufacturer. In brief, T84 cell culture supernatants were thawed, transferred to V-bottom 96-well microtiter plates, and centrifuged for 2.5 min to pellet any precipitates. Supernatants were added in triplicate (one hundred μL per well) to the ELISA assay plate. Seven doses of a known reference standard were added in duplicate, with the highest dose of the standard being 50 ng/mL. Duplicate blank cells served as negative controls. The ELISA assay plate was incubated at 37 °C for 1.5 h. The liquid samples and standards were decanted, and without further washing, freshly prepared biotinylated detection antibody working solution was added to the samples (100 μL/well) and allowed to incubate at 37 °C for 1 h. The ELISA assay plates were washed three times, horseradish peroxidase-conjugate was added (100 μL/well), and incubated at 37 °C for 30 min. The plates were washed five times, after which substrate reagent was added (90 μL/well) and incubated at 37 °C for 15 min in the dark. Stop-solution was added (50 μL/well), and the color reaction was measured by spectrophotometry at 450 nanometers, using a PowerWave microplate reader (BioTek Instruments, Winooski, VT, USA).

### 2.9. Production of Cytokines and Chemokines

After 2 and 24 h of incubation, supernatants were harvested from the T84 cultures, based on previous evidence of detectable cytokine production early versus later in cell cultures [47]. Levels of cytokines and chemokines in the supernatants were quantified using Bio-Plex protein arrays (Bio-Rad Laboratories Inc., Hercules, CA, USA), using xMAP technology (Luminex, Austin, TX, USA): IL-6, IL-8 (CXCL8), IL-10, IL-13, IL-17A, interferon gamma-induced protein 10 (IP-10), MIP-1α (CCL3), MIP-1β (CCL4), granulocyte-macrophage colony-stimulating factor (GM-CSF), and tumor necrosis factor-alpha (TNF-α).

### 2.10. Statistical Analysis

Averages and standard deviations for each data set were calculated using Microsoft Excel (version 2406, Microsoft Corp., Redmond, WA, USA). Statistical analysis was performed using the two-tailed, independent *t*-test. Statistical significance was set at *p* < 0.05 and a high level of statistical significance was set at *p* < 0.01. Trending significance was set at *p* < 0.1.

## 3. Results

### 3.1. Cytokine and Chemokine Levels, 2 h Responses

**Figure 4:**
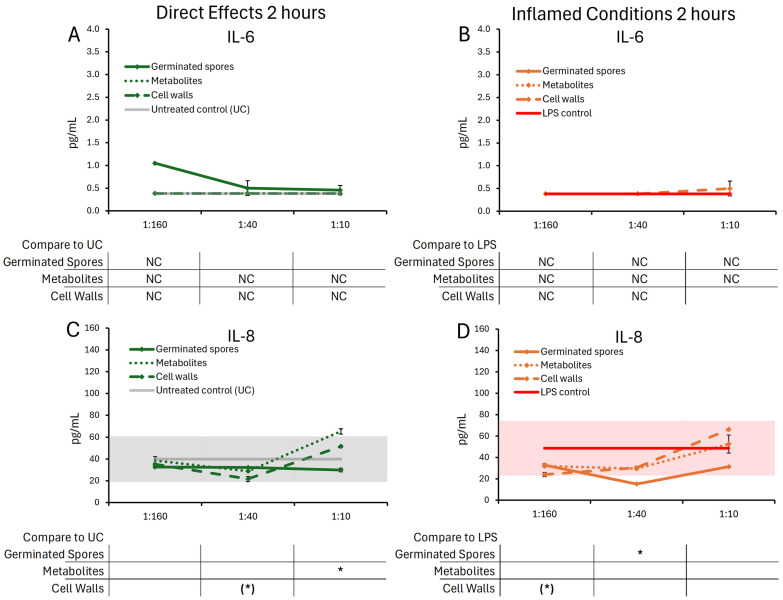
Changes to IL-6 and IL-8 levels in T84 cell cultures after 2 h of exposure to germinated spores, metabolite fraction, and cell wall fraction. The direct effects under normal culture conditions are shown in panels (**A**,**C**). The effects in the context of LPS-induced inflamed conditions are shown in panels (**B**,**D**). The data are shown as the averages ± standard deviation of duplicate data points, compared to control cultures as hexaplicate data points. The averages of untreated control cultures are shown as gray lines, and the standard deviation as a gray shaded area. For IL-6, the lowest dose of the germinated spores had only one data point (not duplicate), and the statistical significance was not calculable (NC); the remaining 2 h data for IL-6 were below levels of significance and also not calculable (NC). The averages of LPS-treated control cultures are shown as red lines, and the standard deviation as a red shaded area. Where the standard deviation is “0”, no shaded area is visible. Levels of statistical significance are shown when comparing the test products to controls, indicated with (*) for *p* < 0.1 and * for *p* < 0.05.

**Figure 5:**
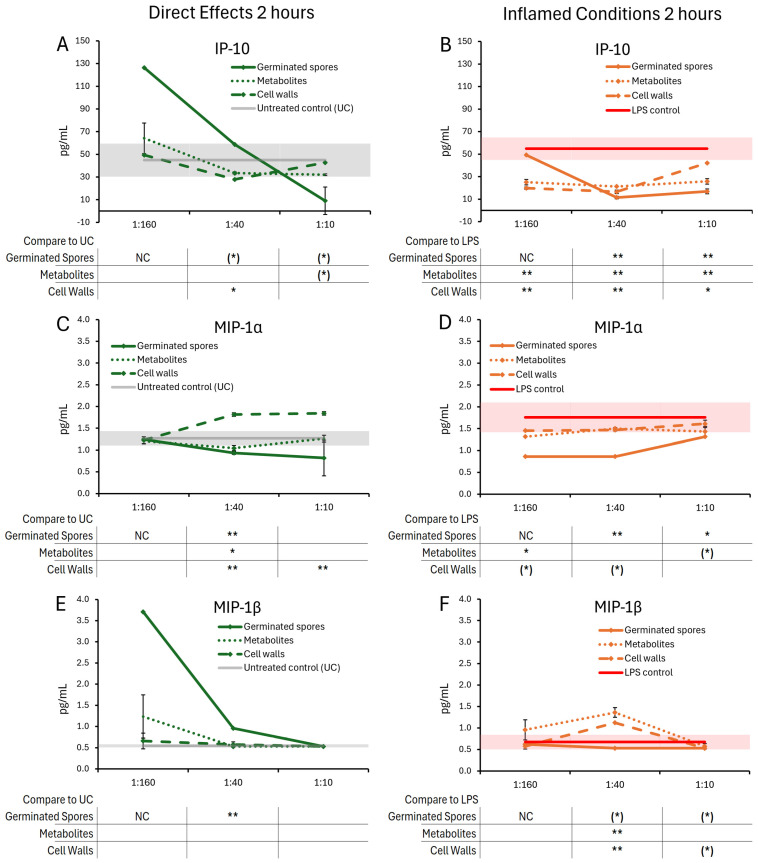
Changes to chemokine levels in T84 cell cultures after 2 h of exposure to germinated spores, metabolite fraction, and cell wall fraction. The direct effects under normal culture conditions are shown in panels (**A**,**C**,**E**). The effects in the context of LPS-induced inflamed conditions are shown in panels (**B**,**D**,**F**). The data are shown as the averages ± standard deviation of duplicate data points, compared to control cultures as hexaplicate data points. The averages of untreated control cultures are shown as gray lines, and the standard deviation as a gray shaded area. For the lowest dose of the germinated spores, only a single data point was available (not duplicate), and the levels of significance were not calculable (NC). Levels of statistical significance are shown when comparing the test products to controls, indicated with (*) for *p* < 0.1, * for *p* < 0.05, and ** for *p* < 0.01.

The cytokine and chemokine levels in T84 gut epithelial cell cultures were evaluated after 2 h of co-culture with the germinated spores, the metabolite fraction, and the cell wall fraction (Figure 4 and Figure 5).

After 2 h of treatment, the test products triggered selective changes to the levels of chemokines IL-8, IP-10, MIP-1α, and MIP-1β (Figure 5), whereas cytokine levels of IL-6 were not significantly affected (Figure 4A,B).

The germinated spores triggered reduced levels of IL-8 (Figure 4C,D) and MIP-1α (Figure 5C,D) under both normal and inflamed culture conditions. The reduction in IL-8 under inflamed conditions reached statistical significance for the middle dose of germinated spores, and the reduction in MIP-1α reached high levels of statistical significance for the middle dose under both normal and inflamed conditions.

Under normal conditions, both the metabolite and cell wall fractions triggered increased IL-8 levels at the highest dose, reaching statistical significance for the metabolite fraction (Figure 4C). Under normal conditions, only the metabolite fraction contributed to the MIP-1α reduction at the middle dose, whereas the cell wall fraction triggered highly significant increased MIP-1α levels at the two higher doses (Figure 5C). Under inflamed conditions, both the metabolite and cell wall fractions contributed to IL-8 and MIP-1α reduction, but their effects were not as robust as the germinated spores (Figure 4D and Figure 5D).

Under normal conditions, the germinated spores triggered a strong biphasic response in IP-10 levels, where the lowest dose triggered an increase in IP-10 production while the highest dose triggered reduced IP-10 levels (Figure 5A). For the lowest dose of the germinated spores, only a single data point was available (not a duplicate), and the levels of significance were not calculable (NC). In contrast, both the metabolite and the cell wall fractions triggered mild decreases in IP-10 levels after 2 h of treatment at the two higher doses, with the highest dose of the metabolite fraction showing a statistical trend and the middle dose of the cell wall fraction reaching statistical significance (Figure 5A). Under inflamed conditions, all three test products triggered comparable decreases in IP-10 levels, reaching high levels of statistical significance at the middle dose (Figure 5B).

Under normal conditions, the germinated spores triggered a strong increase in MIP-1β levels, which was strongest for the two lower doses, reaching a high level of significance for the middle dose (Figure 5E). For the lowest dose of the germinated spores, only a single data point was available (not a duplicate), and the levels of significance were not calculable (NC). Neither the metabolite fraction nor the cell wall fraction triggered significant changes in MIP-1β levels (Figure 5E), indicating that the change induced by the germinated spores interacting with the T84 cells was unique to the living probiotic bacterium. Under inflamed conditions, the germinated spores triggered mildly decreased levels of MIP-1β, reaching a statistical trend for the two higher doses (Figure 5F). In contrast, both the metabolite fraction and the cell wall fraction triggered increased MIP-1β levels, reaching high levels of statistical significance at the middle dose for both fractions (Figure 5F).

### 3.2. Cytokine and Chemokine Levels, 24 h Responses

**Figure 6:**
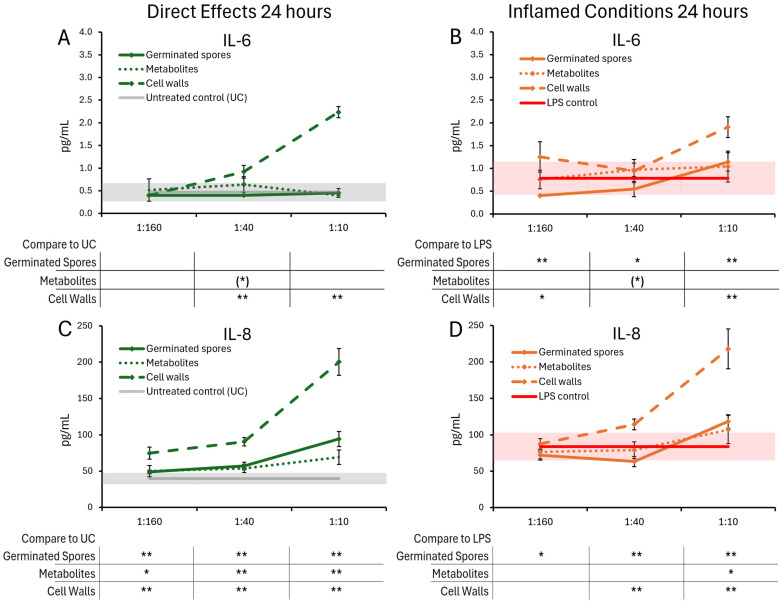
Changes to IL-6 and IL-8 levels in T84 cell cultures after 24 h of exposure to germinated spores, metabolite fraction, and cell wall fraction. The direct effects under normal culture conditions are shown in panels (**A**,**C**). The effects in the context of LPS-induced inflamed conditions are shown in panels (**B**,**D**). The data are shown as the averages ± standard deviation of duplicate data points, compared to control cultures as hexaplicate data points. The averages of untreated control cultures are shown as gray lines, and the standard deviation as a gray shaded area. The averages of LPS-treated control cultures are shown as red lines, and the standard deviation as a red shaded area. Levels of statistical significance are shown when comparing the test products to controls, indicated with (*) for *p* < 0.1, * for *p* < 0.05, and ** for *p* < 0.01.

**Figure 7:**
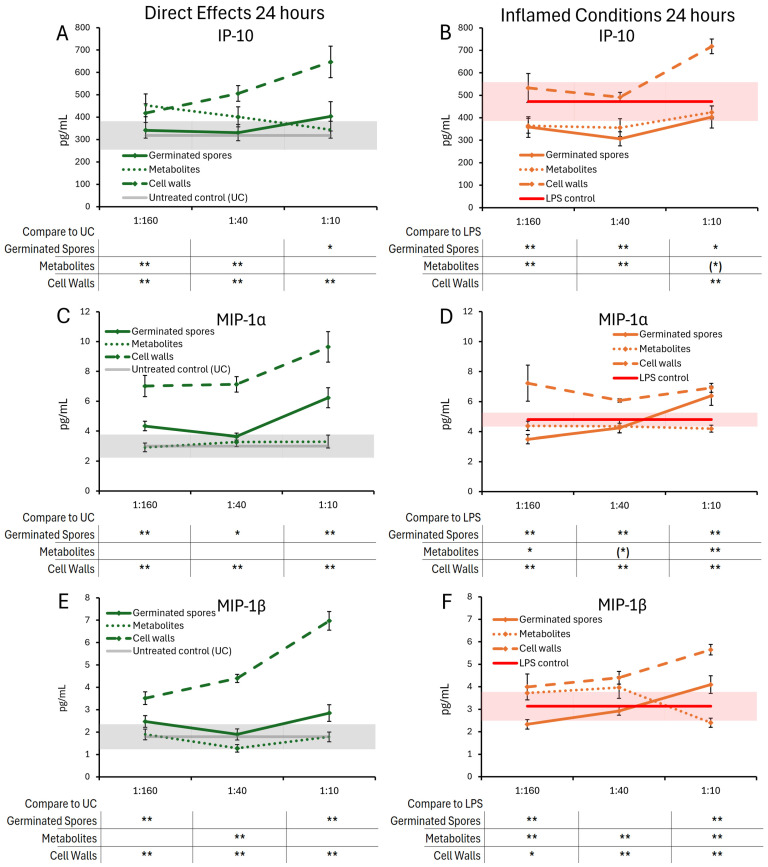
Changes to chemokine levels in T84 cell cultures after 24 h of exposure to germinated spores, metabolite fraction, and cell wall fraction. The direct effects under normal culture conditions are shown in panels (**A**,**C**,**E**). The effects in the context of LPS-induced inflamed conditions are shown in panels (**B**,**D**,**F**). The data are shown as the averages ± standard deviation of duplicate data points, compared to control cultures as hexaplicate data points. The averages of untreated control cultures are shown as gray lines, and the standard deviation as a gray shaded area. The averages of LPS-treated control cultures are shown as red lines, and the standard deviation as red shaded areas. Levels of statistical significance are shown when comparing the test products to controls, indicated with (*) for *p* < 0.1, * for *p* < 0.05, and ** for *p* < 0.01.

The cytokine and chemokine levels in T84 gut epithelial cell cultures were also evaluated 24 h post-treatment with germinated spores, the metabolite fraction, and the cell wall fraction (Figure 6 and Figure 7). Under normal conditions, the germinated spores did not trigger changes to IL-6, but under inflamed conditions, a biphasic reaction was seen, reaching a high level of significance at the lowest and highest doses. The metabolite fraction alone did not contribute to this decrease, as this fraction triggered mild increases in IL-6, reaching statistical trends at the middle dose (Figure 6A,B). Under normal conditions, the germinated spores triggered increased levels of IL-8, which was highly significant for all doses tested (Figure 6C). The metabolite fraction contributed to this change, also reaching high levels of significance for the middle and high doses tested (Figure 6C). Under inflamed conditions, the germinated spores triggered a biphasic effect on IL-8 levels, with an increase at the highest dose, and a decrease at the lower doses (Figure 6D). In contrast, the cell wall fraction triggered increased levels of both IL-6 and IL-8, under both normal and inflamed conditions, reaching high levels of significance for the highest dose (Figure 6A–D).

Under normal conditions, the germinated spores triggered a mild and statistically significant increase in IP-10 levels at the highest dose (Figure 7A) and also triggered highly significant increases in both MIP-1α (Figure 7C) and MIP-1β (Figure 7E).

The metabolite fraction did not contribute to the increased levels of MIP-1α or MIP-1β. In contrast, the middle dose of the metabolite fraction was associated with decreased MIP-1β levels, reaching a high level of significance at this middle dose (Figure 7E).

Under inflamed conditions, both the germinated spores and the metabolite fraction triggered similar reduced levels of IP-10 (Figure 7B), reaching high levels of statistical significance for the two lower doses and a lower level of statistical significance for the highest dose. The germinated spores triggered biphasic changes to MIP-1α and MIP-1β, with decreased levels for the lowest dose and increased levels at the highest dose (*p* < 0.01 for MIP-1α and for MIP-1β). The metabolite fraction triggered reduced levels of MIP-1α (Figure 7D), reaching a high level of statistical significance for the highest dose. The metabolite fraction triggered decreased MIP-1β levels at the highest dose, reaching a high level of statistical significance.

The cell wall fraction triggered increased levels of IP-10, MIP-1α, and MIP-1β under both normal and inflamed conditions, reaching high levels of significance for the highest dose across both conditions (Figure 7A–F).

The following cytokines were not detectable in either 2 h or 24 h culture supernatants: IL-10, IL-13, IL-17A, and GM-CSF. While TNF-α was detectable in untreated control cultures at 24 h, the cytokine was not elevated in cultures treated with any of the three test products.

### 3.3. Wound Healing

**Figure 8:**
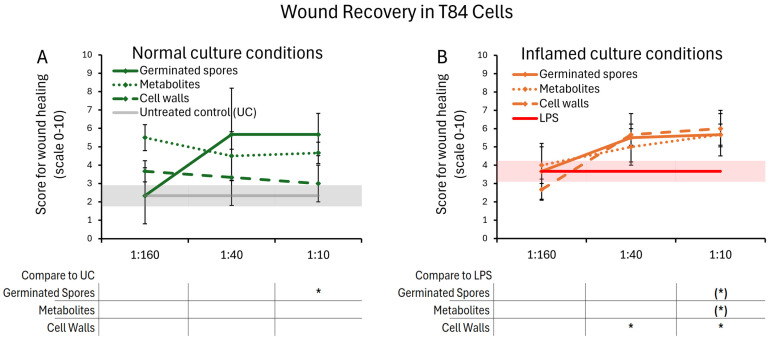
Wound healing after a mechanical scratch under normal (**A**) and LPS-induced inflamed (**B**) culture conditions. Results are shown as averages ± standard deviations for triplicate data points for each dose of germinated spores, metabolite fraction, cell wall fraction, and for each negative and positive control. The average of untreated control cultures is shown as a gray line, and the standard deviation as a gray shaded area. The average of LPS-treated control cultures is shown as a red line, and the standard deviation as a red shaded area. Levels of statistical significance are shown when comparing the test products to controls, indicated with (*) for *p* < 0.1 and * for *p* < 0.05.

The timing of the wound repair of mechanically wounded T84 gut epithelial cells was monitored for five days. Repair was determined by the proportion of recovered cells that had moved into the wound gap to reform an intact cellular monolayer. When comparing wound healing in the absence of test products, it was evident that inflamed cultures recovered slightly faster than cultures under normal culture conditions (comparing the untreated controls in Figure 8A,B). The healing of mechanically wounded T84 gut epithelial cell cultures was accelerated further in the presence of all three test products (Figure 8). This was seen both under normal culture conditions (Figure 8A) and LPS-induced inflamed culture conditions (Figure 8B). The presence of the germinated spores showed a similar recovery under both normal and inflamed culture conditions. In the absence of inflammation, the metabolite fraction supported wound healing better than the cell wall fraction but did not reach statistical significance. Under inflamed conditions, all three test products supported wound healing in a dose-dependent manner and to a similar magnitude, reaching statistical trends for the highest dose for germinated spores and metabolite fraction, and statistical significance for the cell wall fraction at the middle and high doses.

### 3.4. Zonulin Release

**Figure 9:**
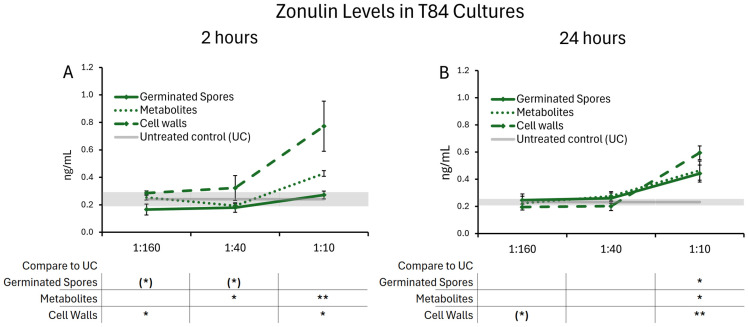
Zonulin release after (**A**) 2 h and (**B**) 24 h of exposure to the probiotic test products. Results are shown as averages ± standard deviations for triplicate data points for germinated spores, the metabolite fraction, and the cell wall fraction. The average of untreated control cultures is shown as a gray line, and the standard deviation as a gray shaded area. Levels of statistical significance are shown when comparing the test products to the untreated controls, indicated with (*) for *p* < 0.1, * for *p* < 0.05, and ** for *p* < 0.01.

The treatment of T84 gut epithelial cells with all test products triggered changes in zonulin release. These changes remained mild, and did not reach levels that would suggest pathological shedding. After 2 h, T84 cells treated with the germinated spores exhibited a decrease in zonulin levels at the two lower doses, suggesting a rapidly increased integrity of the cell layer (Figure 9A), reaching a statistical trend. In contrast, the highest dose of the metabolite and cell wall fraction triggered increased zonulin release. After 24 h, the highest dose of the germinated spores, as well as the metabolite and cell wall fractions, elicited a mild increase in zonulin levels. It is possible that this high dose stimulated a dynamic restructuring of the epithelial monolayer over time to modulate paracellular nutrient transport, requiring further investigation. These data may suggest rapid dynamic changes in monolayer integrity, followed by a restructuring of the cell layer after 24 h.

## 4. Discussion

Gut epithelial cells directly interface with underlying mucosal immune tissue that coordinates localized and systemic immune responses [5]. This study used the human T84 gut epithelial cell model to examine the effects of germinated spores, the metabolite fraction, and the cell wall fraction of the probiotic strain *B. coagulans* JBI-YZ6.3 on communication via cytokine and chemokine secretion, wound healing, and zonulin release. The goal was to examine the impact of exposing gut epithelial tissue to the probiotic strain in the absence and presence of inflammation, in direct extension of our previous work on the effects of this probiotic strain on immune cell activation and modulation of inflammation [40].

The findings presented here demonstrate the effect of the metabolically active *B. coagulans* on the production of cytokines and chemokines by gut epithelial cells, as part of their communication directed at the underlying mucosal immune tissue. The effect of *B. coagulans* JBI-YZ6.3 and its metabolite and cell wall fractions on chemokines was selective and dependent on culture conditions and the duration of co-culture. After 2 h of co-culture, the levels of IL-8 were not significantly affected by the germinated spores under normal culture conditions; however, IL-8 levels were reduced under LPS-inflamed culture conditions, where the reduction was stronger than those seen for either the metabolite fraction or the cell wall fraction. Additionally, under normal conditions, the germinated spores increased IP-10 and MIP-1β levels at the two lower doses, in contrast to either the metabolite or cell wall fractions. At the highest dose of the germinated spores, levels of IP-10 and MIP-1α were reduced, in contrast to an increase in MIP-1α by the cell wall fraction. This suggests that the living probiotic organism conveyed a stronger anti-inflammatory stimulus to the epithelial cells than either of the isolated components.

After 2 h of co-culture under inflamed conditions, the germinated spores had the opposite effect on IP-10, eliciting a decrease in IP-10 at all doses, with contributing effects from both the metabolite and cell wall fractions. Levels of MIP-1α and MIP-1β in the same inflamed cultures were reduced by the germinated spores, in contrast to only marginal effects on MIP-1α and highly significant increases to MIP-1β by the isolated fractions.

After 24 h of co-culture, the cell wall fraction broadly increased the levels of IL-6, IL-8, IP-10, MIP-1α, and MIP-1β. This was expected, as bacterial cell walls are known stimulants of Toll-like receptor-mediated signaling, and 24 h of co-culture of T84 cells with the cell wall fragments was expected to lead to sustained stimulation of the TLR receptors on the T84 cells and increased cytokine production. In contrast, the metabolite fraction and germinated spores triggered only mild changes in the levels of IL-6, IL-8, MIP-1α, and MIP-1β. Under inflamed conditions, the effects of the metabolites and germinated spores on IP-10 levels were striking, as both triggered significant inhibition of LPS-induced increases in IP-10 levels, similar to those exhibited by T84 cells not challenged with LPS.

The metabolites of *B. coagulans* can be absorbed in the gastrointestinal tract and enter the host circulatory system to modulate systemic immune functions [48]. Our previous in vitro study showed that the metabolite fraction from *B. coagulans* JBI-YZ6.3 contributed significantly to its immune-modulating activities and anti-inflammatory effects on human peripheral blood mononuclear cells [40]. Results from this study suggest that metabolites produced by *B. coagulans* JBI-YZ6.3 have anti-inflammatory effects on the gut epithelial cells through the reduction of multiple chemokines under inflamed conditions.

The pro-inflammatory cytokine IL-6, known to initiate acute-phase protein synthesis [49], was not detectable at 2 h of co-culture with T84 cells for any treatment, and multiple other pro-inflammatory cytokines were also absent from both 2 and 24 h of culture. This is relevant for the gut–immune communication at the gut mucosal interface and helps further substantiate the lack of pro-inflammatory activity of the germinated spores. We have previously shown that peripheral blood mononuclear cells, representative of the gut mucosal immune cell community, produce IL-6 when exposed to germinated spores from *B. coagulans* JBI-YZ6.3 [40], suggesting that IL-6 may be produced locally when antigen-presenting immune cells engage with the cell wall of *B. coagulans* by sampling material from the gut lumen through membrane protrusions.

The selective effect of the germinated spores on the chemoattractant signals produced by the T84 cells is a fundamental part of the results presented here. The chemokines produced under normal unstressed culture conditions suggest the T84 cells communicate to recruit antigen-presenting immune cells to the gut tissue. This is in contrast to the reduced chemotactic signals under inflamed conditions, suggesting that when inflamed, the T84 cells are affected by the germinated spores to cause less inflammatory invasion of immune cells capable of causing increased oxidative stress. IL-8 (CXCL8) is secreted by a number of cell types, including intestinal epithelial cells, binding to CXCR1/2 receptors, predominantly expressed on neutrophils, where IL-8-CXCR1/2 binding promotes neutrophil chemotaxis, degranulation, and oxidative burst [50]. IL-8 also promotes angiogenesis and re-epithelialization through its effects on endothelial cells and keratinocytes, respectively [51]. These roles are crucial for clearing pathogens and barrier repair and are tightly regulated, as they are implicated in a number of inflammatory diseases [50]. IP-10 (CXCL10) binds CXCR3 to activate or recruit a variety of cell types, including NK cells, dendritic cells, macrophages, and, less prominently, B cells. Importantly, IP-10 plays a role in inhibiting cell proliferation and angiogenesis, while promoting Th1 immune response [52,53]. MIP-1β (CCL4) acts on CCR5 to recruit cell types similar to those recruited by IP-10 and is involved in functions like wound healing [54,55]. Our findings suggest that, by eliciting chemotactic modulation, the germinated spores of *B. coagulans* JBI-YZ6.3 could selectively promote the recruitment of cell types like monocytes, macrophages, dendritic cells, NK cells, and T cells, while suppressing neutrophil chemotaxis. In contrast, at the same 2 h timepoint under inflamed culture conditions, chemokine levels were reduced compared to the LPS control, suggesting that downstream recruitment of various immune cells could be reduced. This could represent a situation where *B. coagulans* JBI-YZ6.3 modulates the recruitment of certain cell types, favoring increased immunosurveillance under normal conditions, while attenuating this response under inflammatory conditions. Notably, the germinated spores had a stronger effect than the cell walls or the secreted metabolites alone.

**Figure 10:**
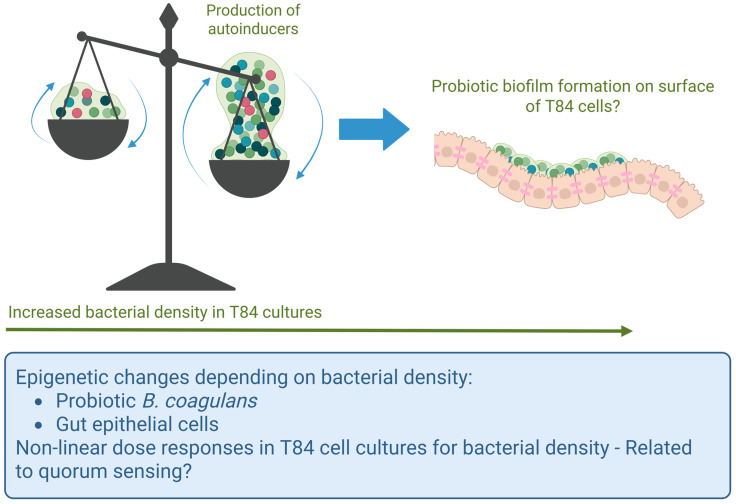
Schematic diagram illustrating the possibility of quorum sensing in relation to the probiotic bacterial density in the co-cultures of T84 gut epithelial cells with *B. coagulans*. At lower bacterial densities, planktonic forms of *B. coagulans* may have produced autoinducers capable of triggering altered gene expression in the T84 cells. In contrast, at the highest dose, *B. coagulans* may have formed biofilms on the apical side of the T84 cells, resulting in a different epigenetic state and a different profile of secreted metabolites affecting the T84 cells.

Our findings showed complex, often biphasic outcomes when T84 cells were treated with escalating doses of germinated spores. One possibility for such biphasic responses is quorum sensing, where population density impacts gene regulation and secreted metabolites, impacting the epigenetic state of both the bacteria and the T84 epithelial cells (Figure 10) [56]. In quorum sensing, bacteria extracellularly secrete autoinducers which, upon reaching a certain density threshold, bind quorum-sensing receptors, which are internalized and act on target genes to regulate physiological functions [57]. While these autoinducers may be species-specific, some can facilitate interspecies crosstalk, including with host cells, and can modulate the host immune responses [58,59,60]. Further investigation is needed to characterize the specific autoinducers of *B. coagulans* and how they might modulate epithelial and immune responses, as they have not been extensively studied. Furthermore, due to the prevalence of biofilm formation on tissue surfaces, where planktonic bacterial forms may be considered a transient form in search of new habitats on which to form biofilms [61], in combination with the strong capacity of *B. coagulans* to adhere to gut epithelial cells [36,57], we suggest that biofilm formation of *B. coagulans* on the T84 cells may have contributed to the non-linear dose responses, specifically at the highest dose.

Our results also demonstrated that exposure to *B. coagulans* supported the integrity of the gut epithelium in several ways. The findings herein demonstrated accelerated wound healing of epithelial cells in both unstressed and LPS-inflamed conditions when treated with *B. coagulans* germinated spores. Previous work has shown that treatment with *B. coagulans* MZY531 promoted the regeneration of damaged intestinal villi [62], and *B. coagulans* strain CGI314 partially ameliorated H_2_O_2_-induced damage to intestinal epithelium [36]. We suggest that each strain may have unique properties; it would be of interest to compare the secreted metabolites from multiple strains alone and in combination in models of epithelial recovery. Gut integrity is critical for human host health, as compromised gut barrier integrity is associated with numerous gastrointestinal issues, wherein leaky gut integrity may play a role in pathogenesis [63]. Our results suggest that *B. coagulans* may be of relevance in the treatment and prevention of gut-integrity-associated pathogenesis by promoting the repair of compromised barriers under both unstressed and inflamed conditions.

Furthermore, the present study demonstrated that exposure to *B. coagulans* JBI-YZ6.3 treatment significantly reduced zonulin release after 2 h. Zonulin regulates tight junctions by reversibly disassembling various zonulin family peptides [19]. A decrease in zonulin release leads to higher levels of tight junction proteins, thereby enhancing tight junction integrity. The intestinal epithelium plays a vital role in both digestion and immunity, and its permeability must be dynamic—permissive enough to allow transport of essential molecules and antigenic sampling, but restrictive enough to prevent passage of toxins and pathogens. Therefore, it is interesting that the highest dose of *B. coagulans* germinated spores triggered a mild increase in zonulin release at 24 h, suggesting increased permeability. While zonulin contributes to this dynamism, dysregulation can lead to compromised barrier integrity, as severe increases in zonulin release are associated with decreased intestinal and immune-related health [64]. More work needs to be done to establish values of zonulin associated with a healthy dynamic gut epithelial function, in contrast to poor gut health, and whether *B. coagulans* JBI-YZ6.3 can support improved nutrient absorption while reducing pathological zonulin release.

While the cell wall fraction is a good control for the physical, non-metabolic activation of gut epithelial cells by the cell wall components binding to pattern-recognition receptors, it would also be of interest for future work to test the effects of ungerminated spores for a more complete understanding of the mechanisms triggered after consuming spores of this probiotic strain. The spore coat has a different chemical composition than the cell wall of germinated spores [65] and would present different patterns and types of envelope material to the human gut epithelial cells, possibly triggering different types and kinetics of gut epithelial cell activation. Challenges include the likely germination if ungerminated spores were added to cell cultures. Also, heat-inactivated ungerminated spores are not ideal for this testing as the heating may denature proteins in the spore coat, so the use of purified superdormant spores would be a suitable approach [66,67].

## 5. Conclusions

In summary, the germinated spores from the probiotic bacterium *B. coagulans* JBI-YZ6.3 directly triggered gut epithelial cells to communicate via chemokine secretion. The germinated spores modulated key aspects of epithelial cell communication and signals to the immune system by reducing inflammatory chemokines, supporting wound healing, and regulating zonulin release. These effects were stronger for the germinated spores compared to the isolated metabolite or cell wall fractions. The data suggest a dual role for germinated spores in promoting epithelial resilience while inhibiting excessive immune activation, especially under inflammatory stress. By selectively enhancing the recruitment of antigen-presenting and regulatory immune cells without provoking neutrophilic inflammation, this probiotic strain may help restore homeostasis along the gut barrier. Future studies investigating co-cultures of *B. coagulans*, T84 epithelial cells, and isolated immune cell types, including monocytes, NK cells, and T cells, are warranted to better understand the ability of *B. coagulans* JBI-YZ6.3 to promote immune cell recruitment in the gut. The results presented here support *B. coagulans* JBI-YZ6.3’s potential clinical probiotic applications in supporting healthy gut epithelial communication to the underlying immune tissue, while also managing gut permeability disorders and chronic inflammatory conditions.

## Data Availability

The original contributions presented in this study are included in the article. Further inquiries can be directed to the corresponding author.

## Abbreviations

The following abbreviations are used in this manuscript:

DMEM	Dulbecco’s modified Eagle’s medium
IL-6	Interleukin-6
IL-8	Interleukin-8
IP-10	Interferon gamma-induced protein 10
MIP-1α	Macrophage inflammatory protein-1 alpha
MIP-1β	Macrophage inflammatory protein-1 beta

## References

[1] Hrncir T. (2022). Gut Microbiota Dysbiosis: Triggers, Consequences, Diagnostic and Therapeutic Options. Microorganisms.

[2] Wardman J.F., Bains R.K., Rahfeld P., Withers S.G. (2022). Carbohydrate-active enzymes (CAZymes) in the gut microbiome. Nat. Rev. Microbiol..

[3] Oliphant K., Allen-Vercoe E. (2019). Macronutrient metabolism by the human gut microbiome: Major fermentation by-products and their impact on host health. Microbiome.

[4] Fan P., Li L., Rezaei A., Eslamfam S., Che D., Ma X. (2015). Metabolites of Dietary Protein and Peptides by Intestinal Microbes and their Impacts on Gut. Curr. Protein Pept. Sci..

[5] McDermott A.J., Huffnagle G.B. (2014). The microbiome and regulation of mucosal immunity. Immunology.

[6] Silva Y.P., Bernardi A., Frozza R.L. (2020). The Role of Short-Chain Fatty Acids from Gut Microbiota in Gut-Brain Communication. Front. Endocrinol..

[7] Ahn J., Hayes R.B. (2021). Environmental Influences on the Human Microbiome and Implications for Noncommunicable Disease. Annu. Rev. Public Health.

[8] Su Q., Liu Q. (2021). Factors Affecting Gut Microbiome in Daily Diet. Front. Nutr..

[9] Sarkar A., Lehto S.M., Harty S., Dinan T.G., Cryan J.F., Burnet P.W.J. (2016). Psychobiotics and the Manipulation of Bacteria-Gut-Brain Signals. Trends Neurosci..

[10] Chen Y.Y., Chen D.Q., Chen L., Liu J.R., Vaziri N.D., Guo Y., Zhao Y.Y. (2019). Microbiome-metabolome reveals the contribution of gut-kidney axis on kidney disease. J. Transl. Med..

[11] Lavelle A., Sokol H. (2020). Gut microbiota-derived metabolites as key actors in inflammatory bowel disease. Nat. Rev. Gastroenterol. Hepatol..

[12] De Angelis M., Garruti G., Minervini F., Bonfrate L., Portincasa P., Gobbetti M. (2019). The Food-gut Human Axis: The Effects of Diet on Gut Microbiota and Metabolome. Curr. Med. Chem..

[13] Agus A., Clément K., Sokol H. (2021). Gut microbiota-derived metabolites as central regulators in metabolic disorders. Gut.

[14] Parsaei M., Sarafraz N., Moaddab S.Y., Ebrahimzadeh Leylabadlo H. (2021). The importance of *Faecalibacterium prausnitzii* in human health and diseases. New Microbes New Infect..

[15] Singh V., Lee G., Son H., Koh H., Kim E.S., Unno T., Shin J.H. (2023). Butyrate producers, “The Sentinel of Gut”: Their intestinal significance with and beyond butyrate, and prospective use as microbial therapeutics. Front. Microbiol..

[16] Schepici G., Silvestro S., Bramanti P., Mazzon E. (2019). The Gut Microbiota in Multiple Sclerosis: An Overview of Clinical Trials. Cell Transplant..

[17] Feldman G.J., Mullin J.M., Ryan M.P. (2005). Occludin: Structure, function and regulation. Adv. Drug Deliv. Rev..

[18] Furuse M., Takai Y. (2021). Recent advances in understanding tight junctions. Fac. Rev..

[19] Kuo W.T., Odenwald M.A., Turner J.R., Zuo L. (2022). Tight junction proteins occludin and ZO-1 as regulators of epithelial proliferation and survival. Ann. N. Y. Acad. Sci..

[20] Sturgeon C., Fasano A. (2016). Zonulin, a regulator of epithelial and endothelial barrier functions, and its involvement in chronic inflammatory diseases. Tissue Barriers.

[21] Veres-Székely A., Szász C., Pap D., Szebeni B., Bokrossy P., Vannay Á. (2023). Zonulin as a Potential Therapeutic Target in Microbiota-Gut-Brain Axis Disorders: Encouraging Results and Emerging Questions. Int. J. Mol. Sci..

[22] Fasano A. (2011). Zonulin and its regulation of intestinal barrier function: The biological door to inflammation, autoimmunity, and cancer. Physiol. Rev..

[23] Hill C., Guarner F., Reid G., Gibson G.R., Merenstein D.J., Pot B., Morelli L., Canani R.B., Flint H.J., Salminen S. (2014). The International Scientific Association for Probiotics and Prebiotics consensus statement on the scope and appropriate use of the term probiotic. Nat. Rev. Gastroenterol. Hepatol..

[24] Chaudhari A., Dwivedi M.K. (2022). Probiotics in the Prevention and Management of Human Diseases.

[25] Marteau P.R. (2002). Probiotics in clinical conditions. Clin. Rev. Allergy Immunol..

[26] Gionchetti P., Rizzello F., Venturi A., Brigidi P., Matteuzzi D., Bazzocchi G., Poggioli G., Miglioli M., Campieri M. (2000). Oral bacteriotherapy as maintenance treatment in patients with chronic pouchitis: A double-blind, placebo-controlled trial. Gastroenterology.

[27] Weizman Z., Asli G., Alsheikh A. (2005). Effect of a probiotic infant formula on infections in child care centers: Comparison of two probiotic agents. Pediatrics.

[28] D’Souza A.L., Rajkumar C., Cooke J., Bulpitt C.J. (2002). Probiotics in prevention of antibiotic associated diarrhoea: Meta-analysis. BMJ.

[29] Cheng L.H., Liu Y.W., Wu C.C., Wang S., Tsai Y.C. (2019). Psychobiotics in mental health, neurodegenerative and neurodevelopmental disorders. J. Food Drug Anal..

[30] Stanton C., Desmond C., Coakley M., Collins J.V., Fitzgerald G.F., Ross R.P. (2003). Challenges facing development of probiotic-containing functional foods. Handbook of Fermented Functional Foods.

[31] Ayichew T., Belete A., Alebachew T., Tsehaye H., Berhanu H., Minwuyelet A. (2017). Bacterial probiotics their importances and limitations: A review. J. Nutr. Health Sci..

[32] Keller D., Verbruggen S., Cash H., Farmer S., Venema K. (2019). Spores of *Bacillus coagulans* GBI-30, 6086 show high germination, survival and enzyme activity in a dynamic, computer-controlled in vitro model of the gastrointestinal tract. Benef. Microbes.

[33] Bora P.S., Puri V., Bansal A.K. (2009). Physicochemical Properties and Excipient Compatibility studies of Probiotic *Bacillus coagulans* Spores. Sci. Pharm..

[34] Bomko T.V., Nosalskaya T.N., Kabluchko T.V., Lisnyak Y.V., Martynov A.V. (2017). Immunotropic aspect of the *Bacillus coagulans* probiotic action. J. Pharm. Pharmacol..

[35] Koh Y.C., Chang Y.C., Lin W.S., Leung S.Y., Chen W.J., Wu S.H., Wei Y.S., Gung C.L., Chou Y.C., Pan M.H. (2024). Efficacy and Mechanism of the Action of Live and Heat-Killed *Bacillus coagulans* BC198 as Potential Probiotic in Ameliorating Dextran Sulfate Sodium-Induced Colitis in Mice. ACS Omega.

[36] Mazhar S., Simon A., Khokhlova E., Colom J., Leeuwendaal N., Deaton J., Rea K. (2024). In vitro safety and functional characterization of the novel *Bacillus coagulans* strain CGI314. Front. Microbiol..

[37] Chang X., Kang M., Shen Y., Yun L., Yang G., Zhu L., Meng X., Zhang J., Su X. (2021). *Bacillus coagulans* SCC-19 maintains intestinal health in cadmium-exposed common carp (*Cyprinus carpio* L.) by strengthening the gut barriers, relieving oxidative stress and modulating the intestinal microflora. Ecotoxicol. Environ. Saf..

[38] Cao J., Zhiming Y., Wenyin L., Jianxin Z., Hao Z., Qixiao Z., Wei C. (2020). Probiotic characteristics of *Bacillus coagulans* and associated implications for human health and diseases. J. Funct. Foods.

[39] Mu Y., Cong Y. (2019). *Bacillus coagulans* and its applications in medicine. Benef. Microbes.

[40] Iloba I., McGarry S.V., Yu L., Cruickshank D., Jensen G.S. (2023). Differential Immune-Modulating Activities of Cell Walls and Secreted Metabolites from Probiotic *Bacillus coagulans* JBI-YZ6.3 under Normal versus Inflamed Culture Conditions. Microorganisms.

[41] Jensen G.S., Benson K.F., Carter S.G., Endres J.R. (2010). GanedenBC30 cell wall and metabolites: Anti-inflammatory and immune modulating effects in vitro. BMC Immunol..

[42] Benson K.F., Redman K.A., Carter S.G., Keller D., Farmer S., Endres J.R., Jensen G.S. (2012). Probiotic metabolites from *Bacillus coagulans* GanedenBC30™ support maturation of antigen-presenting cells in vitro. World J. Gastroenterol..

[43] Devriese S., Van den Bossche L., Van Welden S., Holvoet T., Pinheiro I., Hindryckx P., De Vos M., Laukens D. (2017). T84 monolayers are superior to Caco-2 as a model system of colonocytes. Histochem. Cell Biol..

[44] Mallegol J., Van Niel G., Lebreton C., Lepelletier Y., Candalh C., Dugave C., Heath J.K., Raposo G., Cerf-Bensussan N., Heyman M. (2007). T84-intestinal epithelial exosomes bear MHC class II/peptide complexes potentiating antigen presentation by dendritic cells. Gastroenterology.

[45] Zhang Y., Gandhi N.N. (2023). Complete Genomic Sequence of *Bacillus coagulans* Strain JBI-YZ6.3: A Natural Spore-Forming Isolate from Food-Grade Tapioca Starch. Microbiol. Resour. Announc..

[46] Zhang Y., Overbeck T.J., Skebba V.L.P., Gandhi N.N. (2024). Genomic and Phenotypic Safety Assessment of Probiotic *Bacillus coagulans* Strain JBI-YZ6.3. Probiotics Antimicrob. Proteins.

[47] McGarry S.V., Yu L., Cruickshank D., Iloba I., Jensen G.S. (2024). Immune Activation by a Nutraceutical Blend: Rapid Increase in Immune-Modulating Cytokines, Followed by Induction of Anti-Inflammatory and Restorative Biomarkers. Nutraceuticals.

[48] O’Riordan K.J., Collins M.K., Moloney G.M., Knox E.G., Aburto M.R., Fülling C., Morley S.J., Clarke G., Schellekens H., Cryan J.F. (2022). Short chain fatty acids: Microbial metabolites for gut-brain axis signalling. Mol. Cell Endocrinol..

[49] Tanaka T., Narazaki M., Kishimoto T. (2014). IL-6 in inflammation, immunity, and disease. Cold Spring Harb. Perspect. Biol..

[50] Ha H., Debnath B., Neamati N. (2017). Role of the CXCL8-CXCR1/2 Axis in Cancer and Inflammatory Diseases. Theranostics.

[51] Matsushima K., Yang D., Oppenheim J.J. (2022). Interleukin-8: An evolving chemokine. Cytokine.

[52] Elemam N.M., Talaat I.M., Maghazachi A.A. (2022). CXCL10 Chemokine: A Critical Player in RNA and DNA Viral Infections. Viruses.

[53] Liu M., Guo S., Hibbert J.M., Jain V., Singh N., Wilson N.O., Stiles J.K. (2011). CXCL10/IP-10 in infectious diseases pathogenesis and potential therapeutic implications. Cytokine Growth Factor. Rev..

[54] Chang T.T., Chen J.W. (2016). Emerging role of chemokine CC motif ligand 4 related mechanisms in diabetes mellitus and cardiovascular disease: Friends or foes?. Cardiovasc. Diabetol..

[55] Cook D.N. (1996). The role of MIP-1 alpha in inflammation and hematopoiesis. J. Leukoc. Biol..

[56] Bressuire-Isoard C., Broussolle V., Carlin F. (2018). Sporulation environment influences spore properties in Bacillus: Evidence and insights on underlying molecular and physiological mechanisms. FEMS Microbiol. Rev..

[57] Deng Z., Luo X.M., Liu J., Wang H. (2020). Quorum Sensing, Biofilm, and Intestinal Mucosal Barrier: Involvement the Role of Probiotic. Front. Cell Infect. Microbiol..

[58] Wu L., Luo Y. (2021). Bacterial Quorum-Sensing Systems and Their Role in Intestinal Bacteria-Host Crosstalk. Front. Microbiol..

[59] Bandyopadhaya A., Tsurumi A., Rahme L.G. (2017). NF-κBp50 and HDAC1 Interaction Is Implicated in the Host Tolerance to Infection Mediated by the Bacterial Quorum Sensing Signal 2-Aminoacetophenone. Front. Microbiol..

[60] Li H., Li X., Ai Q., Tan L. (2022). Autoinducer-2 promotes *Pseudomonas aeruginosa* PAO1 acute lung infection via the IL-17A pathway. Front. Microbiol..

[61] Motta J.P., Wallace J.L., Buret A.G., Deraison C., Vergnolle N. (2021). Gastrointestinal biofilms in health and disease. Nat. Rev. Gastroenterol. Hepatol..

[62] Zhao Z., Sun M., Cui X., Chen J., Liu C., Zhang X. (2023). *Bacillus coagulans* MZY531 alleviates intestinal mucosal injury in immunosuppressive mice via modulating intestinal barrier, inflammatory response, and gut microbiota. Sci. Rep..

[63] Chelakkot C., Ghim J., Ryu S.H. (2018). Mechanisms regulating intestinal barrier integrity and its pathological implications. Exp. Mol. Med..

[64] Tajik N., Frech M., Schulz O., Schälter F., Lucas S., Azizov V., Dürholz K., Steffen F., Omata Y., Rings A. (2020). Targeting zonulin and intestinal epithelial barrier function to prevent onset of arthritis. Nat. Commun..

[65] Salton M.R.J., Marshall B. (1959). The Composition of the Spore Wall and the Wall of Vegetative Cells of *Bacillus subtilis*. Microbiology.

[66] Ghosh S., Setlow P. (2009). Isolation and characterization of superdormant spores of Bacillus species. J. Bacteriol..

[67] Delbrück A.I., Zhang Y., Hug V., Trunet C., Mathys A. (2021). Isolation, stability, and characteristics of high-pressure superdormant *Bacillus subtilis* spores. Int. J. Food Microbiol..

